# Jumping Through Hoops: Community Care Clinician and Staff Experiences Providing Primary Care to Rural Veterans

**DOI:** 10.1007/s11606-023-08126-2

**Published:** 2023-06-20

**Authors:** Mary Patzel, Chrystal Barnes, NithyaPriya Ramalingam, Rose Gunn, Erin S. Kenzie, Sarah S. Ono, Melinda M. Davis

**Affiliations:** 1 Oregon Rural Practice-Based Research Network, Oregon Health & Science University, 3181 SW Sam Jackson Park Rd, Mail Code: L222, Portland, OR 97239 USA; 2 OCHIN, Inc., Portland, OR USA; 3 Department of Veterans Affairs Office of Rural Health, Veteran Rural Health Resources Center, Portland, OR USA; 4 Department of Psychiatry, Oregon Health & Science University, Portland, OR USA; 5 Department of Family Medicine and OHSU-PSU School of Public Health, Oregon Health & Science University, Portland, OR USA

**Keywords:** Primary care, Rural health services, Veterans, Equitable access

## Abstract

**Background:**

The 2019 VA Maintaining Systems and Strengthening Integrated Outside Networks Act, or MISSION Act, aimed to improve rural veteran access to care by expanding coverage for services in the community. Increased access to clinicians outside the US Department of Veterans Affairs (VA) could benefit rural veterans, who often face obstacles obtaining VA care. This solution, however, relies on clinics willing to navigate VA administrative processes.

**Objective:**

To investigate the experiences rural, non-VA clinicians and staff have while providing care to rural veterans and inform challenges and opportunities for high-quality, equitable care access and delivery.

**Design:**

Phenomenological qualitative study.

**Participants:**

Non-VA-affiliated primary care clinicians and staff in the Pacific Northwest.

**Approach:**

Semi-structured interviews with a purposive sample of eligible clinicians and staff between May and August 2020; data analyzed using thematic analysis.

**Key Results:**

We interviewed 13 clinicians and staff and identified four themes and multiple challenges related to providing care for rural veterans: (1) Confusion, variability and delays for VA administrative processes, (2) clarifying responsibility for dual-user veteran care, (3) accessing and sharing medical records outside the VA, and (4) negotiating communication pathways between systems and clinicians. Informants reported using workarounds to combat challenges, including using trial and error to gain expertise in VA system navigation, relying on veterans to act as intermediaries to coordinate their care, and depending on individual VA employees to support provider-to-provider communication and share system knowledge. Informants expressed concerns that dual-user veterans were more likely to have duplication or gaps in services.

**Conclusions:**

Findings highlight the need to reduce the bureaucratic burden of interacting with the VA. Further work is needed to tailor structures to address challenges rural community providers experience and to identify strategies to reduce care fragmentation across VA and non-VA providers and encourage long-term commitment to care for veterans.

## INTRODUCTION

Nearly one quarter of veterans living in the USA reside in rural areas, 4.7 million in total.^1^ Rural veterans have been shown to have lower health-related quality of life scores than urban veterans^2^ and are more likely to have lower educational attainment, lower employment rates, and higher rates of disability. Rural veterans are also more likely to be older, not employed, living with a disability, and living in poverty when compared to rural non-Veterans.^3^ Fifty-five percent of rural veterans enrolled in Department of Veterans Affairs (VA) health care are over the age of 65 and are more likely to have more complex medical conditions than Veterans living in urban areas.^4,5^

Rural Veterans are more likely to be enrolled in VA health care than their urban counterparts (58% vs. 38%), despite fewer than 20% of VA hospitals being located in rural areas.^1,6^ While rural veterans rely heavily on VA coverage for health care, VA locations are limited and rural Veterans often face significant challenges accessing and navigating health care.^7–9^ Rural veteran age and health status can further complicate the challenges of geographic distance.^8,10,11^

The VA contributes significant resources to address the care needs of rural veterans.^12^ Two main strategies include maintaining VA-owned-and-operated community-based outpatient clinics and providing opportunities for veterans to receive care from non-VA clinicians under certain qualifying circumstances. This system of paying non-VA clinicians to care for VA-covered veterans is commonly referred to as “community care.”^13^ Community care has supplemented VA-provided health care since World War I, but veteran eligibility for community care has expanded significantly since 2014.^12–15^ In addition, the 2019 VA Maintaining Systems and Strengthening Integrated Outside Networks Act, or MISSION Act, further expanded availability of community care services via changes to eligibility requirements.^16,17^ The number of veterans eligible for community care rose steeply between 2014 and 2020, and 2.3 million veterans were authorized to use outside care via community care agreements in 2020.^12^

While increased access to community care can benefit rural veterans, this solution relies on a widespread network of non-VA clinics willing to offer care and navigate VA approval and administrative processes. Despite an increasing number of veterans eligible for and using community care or both VA-based services and community care, the research exploring non-VA clinician and clinic staff experiences caring for veterans is limited.^18–21^ The purpose of this study was to understand the experiences non-VA clinicians and staff in rural settings have while providing care to veterans and to inform opportunities for high-quality, sustainable, and equitable care access and delivery.

## METHODS

### Study Design

A multidisciplinary study team conducted this qualitative study using a phenomenological approach, which included studying the shared lived experiences of non-VA-affiliated primary care clincians and staff members.^24^ Semi-structured interviews were conducted between May and August 2020. This study was approved by the VA Portland Health Care System (VAPORHCS)/Oregon Health and Science University (OHSU) joint IRB (eIRB#20,843).

### Setting

Qualitative analysts interviewed a purposive sample of non-VA affiliated primary care clinicians and staff members who provide care for rural veterans in the Pacific Northwest (i.e., Oregon, Washington, Idaho).

### Data Collection and Analysis

We prioritized interviews with representatives from rural clinics, as identified by the Oregon Office of Rural Health and/or Rural Urban Continuum Area codes.^25,26^ While our focus was on facilities in rural communities, some of the interviews were conducted with staff from micropolitan clinics that served a wide catchment area that included adjacent rural communities.

Our team developed a 17-question semi-structured interview guide focused on the following domains: experiences caring for rural veterans, barriers and recommendations to improve community care, impacts of the VA MISSION Act, and impacts from COVID-19. These guides were developed by our team prior to data collection and reviewed by the study’s Rural Veteran Advisory Board to clarify language and flow. This Advisory Board includes 10 stakeholders, including rural veterans, Veteran Service Officers, and VA and non-VA primary care clinicians and clinic staff members. Guides were iteratively refined during data collection based on the initial review of transcripts.

To reach a variety of perspectives, we purposively recruited clinics based on their location, number of Veterans served, and clinic and participant role.^27^ We reached out to a key clinic staff member, such as a clinic manager or medical director to recruit staff via email or phone. Out of the 45 clinics we contacted, 12 agreed to participate, at which point we reached data saturation, the point at which themes repeat or recoccur, and ended recruitment.^28^ All interviews were conducted from May to August 2020 by an experienced qualitative analyst (MP and ESK) via videoconference or telephone. Informants provided verbal consent prior to the start of each interview. Interviews lasted 30–60 min in duration and were recorded and transcribed using a third-party service. Transcribed interviews were then validated, de-identified by a study team member, and uploaded to ATLAS.ti (Version 8.0, Scientific Software Development GmbH) for data management and analysis.

Concurrent with data collection, the data were analyzed from June to October 2020 using thematic analysis.^29^ A codebook was developed using a combination of inductive and deductive coding and included a priori codes based on the research aims as well as novel codes drawing directly from the data. The codebook was tested on transcripts by three primary coders (ESK, NR, MP) and revised iteratively in an initial trial coding phase during meetings with the full analytic team, which included the primary analysts, the study PI (MMD), and a veteran stakeholder with medical training. After the final codebook was created, remaining transcripts were coded by one of two analysts (ESK and NR). A subset of these transcripts were coded by a second analyst (MP) for reliability. Emergent themes were identified through a dialogue-based process in which the team prioritized codes for closer analysis and discussed data queries. Data and emergent themes were shared with the Rural Veteran Advisory Board in Fall 2020 to check findings and to refine emergent themes.^30^

## RESULTS

**Fig. 1:**
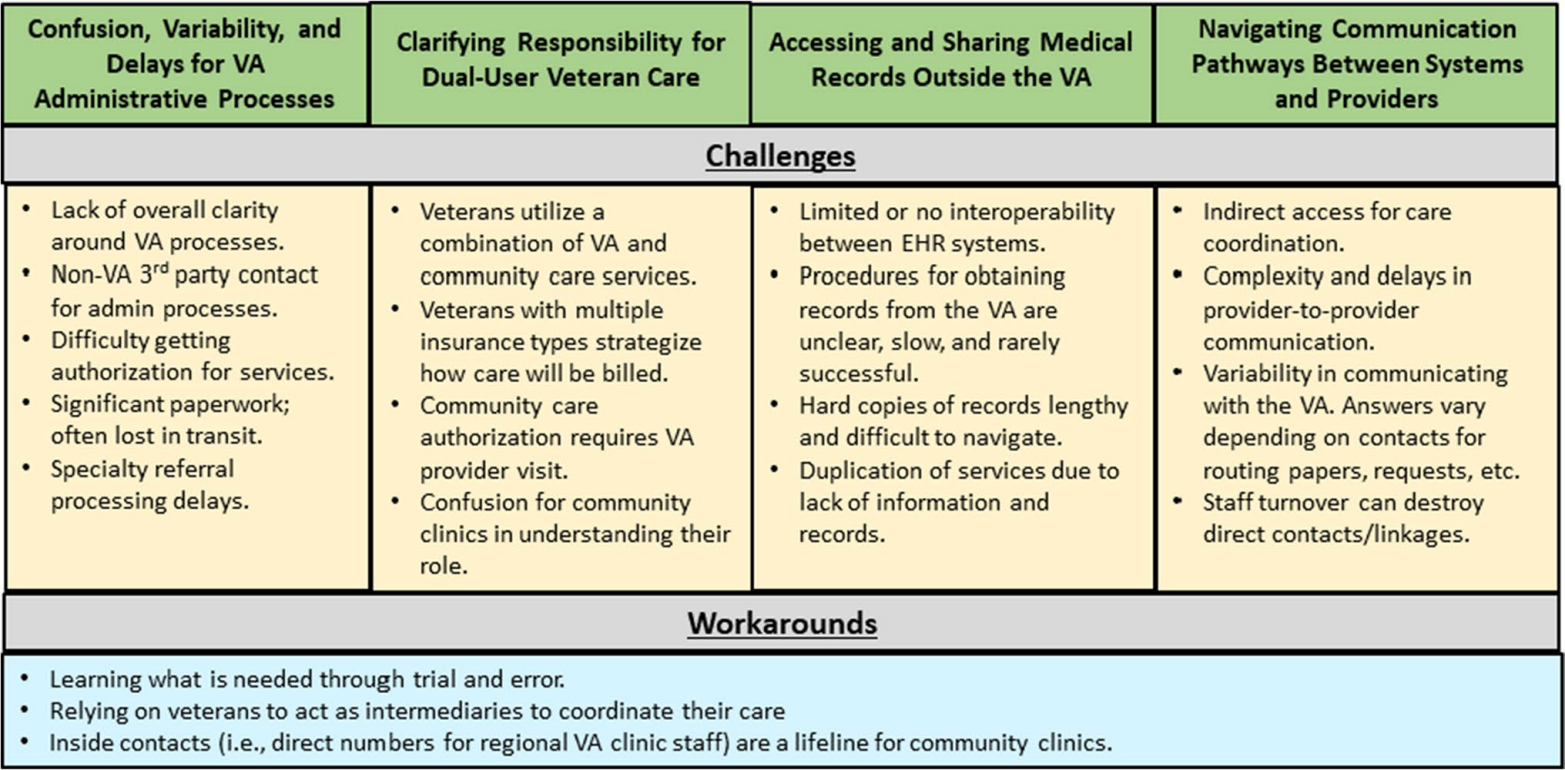
Summary of qualitative themes

We interviewed 13 clinician and clinic staff informants. Over two-thirds of informants were clinicians (i.e., MD, DO, PA, NP); the remainder included clinic staff working in administrative roles. Two informants were Veterans. Informants worked at private and hospital-affiliated clinics; the majority of the clinics (69%) were designated as Federally Certified Rural Health Clinics, all located in the Pacific Northwest region (i.e., Washington, Oregon, Idaho). Four main themes emerged from clinicians and clinic staff data related to providing care for rural veterans: (1) confusion, variability, and delays for VA administrative processes; (2) clarifying responsibility for dual-user veteran care, (3) accessing and sharing medical records outside the VA, and (4) negotiating communication pathways between systems and providers. Themes are summarized below in Fig. 1.

Informants reported using workarounds to combat challenges, including using trial and error to gain expertise navigating VA systems, relying on veterans to act as intermediaries to coordinate their own care, and depending on individual VA employees to support provider-to-provider communication pathways and share system knowledge. Informants expressed concerns that dual-user rural veterans, those who are eligible for and utilize both VA-based services and community care, were more likely to have duplication or gaps in services due to these challenges.

### Confusion, variability, and delays for VA administrative processes

Participants described a variety of VA administrative processes that differ from those required for non-veteran patients and non-VA insurers. In their work with civilian insurers, participants typically contact a health plan to confirm authorization for services on a patient’s behalf. In contrast, VA insurance requires the veteran to consult with the VA Office of Community Care on a regular basis to secure prior authorization for primary care services rendered outside of the VA. Any needed service not included in the initial authorization needs to be approved for secondary authorization. TriWest is contracted to manage community care for the Pacific Northwest region, often adding another party to administrative processes. Participants identified understanding processes and protocols set by the VA and/or TriWest as a primary challenge to navigating the VA and caring for veteran patients, with one participant describing it as a “black box” (Clinician 10). Nearly all participants expressed some degree of confusion about VA processes, and this lack of clarity permeated almost all aspects of care for rural veterans in community settings.

One particular process that caused both confusion and delay was the authorization of primary care services through community care consultations. Several participants noted a lack of clarity and high degree of variability for authorizations, particularly in ensuring the full spectrum of primary care services is covered in the initial authorization. One clinic staff member noted the importance of patient self-advocacy when navigating challenges in authorizations:

*I would say that's probably one out of five veterans that gets [all appropriate primary care services] included in the initial authorization . . . so it's beneficial to those who are a good advocate for themselves, but those individuals that are left without anyone to hold their hand, they're left with longer processing times in the long run because we have to do more for them*. -Staff member (02)

Participants also reported that paperwork submitted to the VA—for purposes such as prescribing medications, securing care authorizations and specialty referrals, and reimbursement—were subject to long processing times and were often misplaced or reported as lost. The VA’s reliance on communication systems such as mail or fax were described as unreliable and led to feelings of helplessness on the part of clinic staff. One clinic staff member described these paperwork challenges in the context of prescribing certain classes of medications for VA patients:

*It seems like they use the oldest methods; like, for a Schedule II drug, a written prescription, they have to be mailed to [the VA]—mailed—snail mailed, and they get lost . . . all the time. We've had this veteran—it's the same thing every month. It gets lost probably three months out of six. And you ask, you call the VA, you're frustrated, you ask, "What are we doing wrong? What can be done to prevent this?”* –Staff member (02)

Processing delays for specialist referrals from community clinicians were of particular concern to participants, especially in time-sensitive cases, such as cancer care, behavioral health, physical therapy, and hospice care. Participants described how the standard turnaround time of 14 days for specialty care referrals caused delayed care and frustration to themselves and the veterans. Several clinicians reported significantly longer processing delays of up to 2 to 3 months for referral authorizations. A number of participants expressed concerns that delays in specialist care contributed to worse health outcomes for patients.

Participants noted that knowledge about difficult administrative processes was often gained through trial and error as issues arose. Multiple participants reported that they were only made aware of policy or workflow changes when they received an error notification or request denial.

### Clarifying Responsibility for Dual-User Veteran Care

Several participants shared that veteran patients typically received a combination of VA and community care services. They described how these dual-users often retained both a VA and non-VA primary care doctor in order to strategically access care in both systems. Participants noted that for veterans with multiple types of insurance, VA coverage limitations require them to carefully plan how their care will be billed, as illustrated in the following quote:

*A lot of my patients . . . use their Medicare benefits until financially they can't handle paying that 20%, and then they come back and a lot of times they'll selectively say, "Okay, this will be a VA visit because we're going to order some stuff from the VA. Next visit will be a Medicare visit and we'll do this other stuff on Medicare so I can actually get the care that I need." And that's not right.* –Clinician and veteran (04)

Participants expressed confusion understanding their role in caring for veterans who receive care at both VA and non-VA provider sites. Participants described how veteran patients often acted as intermediaries between their providers as a common workaround to combat fragmented care across non-VA and VA settings and clinicians. A number of participants noted opportunities for improvement to streamline continuity of care by optimizing communications between the VA, veteran patients, and non-VA clinicians.

### Accessing and Sharing Medical Records Outside the VA

Participants described accessing and sharing medical records as challenging when working with the VA. Participants described numerous challenges sending and receiving veteran patient medical records with the VA due to bureaucratic hurdles and the challenges with the different EHR systems. Procedures for obtaining records from the VA were described as unclear, slow, and rarely successful. Some participants noted that requests for medical records from the VA can take up to 6 months, if they are fulfilled at all. Participants who received hard copy records from the VA said that the records were lengthy and difficult to navigate. These challenges were summarized by one informant who expressed the following:

*I've been in practice now for six years and I can very clearly tell you that I have seen the records from a VA patient, the actual historical record of whatever that I requested, three times . . . and the records are four to 500 pages long* -Clinician (07)

Some participants described how certain EHRs have the ability to receive patient records electronically through the VA EHR, but the integration remains limited. Only one provider, whose clinic had an uncommonly close relationship with the VA, mentioned receiving records electronically. However, this same participant relayed a story about a patient who had to travel a hundred miles to physically sign a paper enabling this integration.

The burdensome nature of record sharing often leads to duplication of services, fragmentation, and delayed care, according to participants. One clinician described these challenges in relation to veteran patient labs:

*I don't get a copy of those [lab] results. Then I'm following [the veteran] and I'm supposed to be the one taking care of them. [. . .] And it's really hard to practice standard of care and provide the best care possible when there are these pieces out there. And I know there's data, but I don't know what it is. I can't be a good steward of your resources [in this model]. I don't want to repeat labs that have been done.. . . So I would like to see a little bit more two-way street, because I really do want to take care of these patients.*

-Clinician (12)

### Negotiating Communication Pathways Between Systems and Providers

Participants noted that all communications related to VA-approved care went through what was perceived to be a centralized VA channel, typically a centralized call center, sometimes creating a barrier for clinic staff wanting to directly reach other providers involved in veteran care. Clinicians and staff members expressed frustration in the complexity of and delays in provider-to-provider communication during VA-approved care. Providers noted that communication and records passed through the VA, even for two non-VA providers, which reduced their ability to communicate directly with each other. Providers and staff also described challenges connecting to VA providers, as all calls were routed through centralized VA Medical Center operators, subject to delays as illustrated by the following quote:

*I think the biggest problem is that there’s a separation between the local VA clinic and the great VA and us [. . .] It’s literally extremely difficult to get in contact with the VA because all of their phone calls are routed through [the large urban VA Medical Center]. If I want to get a patient’s medical records from there, I have to talk to medical records [the large urban VA Medical Center], not the local VA*. -Clinician (01)

In addition to challenges connecting directly with providers and clinic staff, informants described challenges and variability communicating with the VA system. Interviewees noted that they received different answers to their questions about processes when speaking with different staff at the VA. Several participants stated they received contradictory information about the protocol for VA interaction. One staff member (02) reported “large inconsistencies on the information given on the proper routes and channels to get care done correctly without major wait times,” during different conversations with VA staff. Interviewees also recounted challenges being kept up to date when processes or policies changed at the VA. For example, few informants were aware of the MISSION Act and how this legislation may impact their veteran population or care processes.

Informants discussed how success often relies on utilizing individual VA employees to support provider-to-provider communication pathways and share inside knowledge. Participants noted that they maintained personal contact information for local VA-clinic staff in order to bypass the official VA phone tree. If possible, community clinic informants contacted a particular person with whom they had an established relationship for assistance navigating the system. While participants described how these inside contacts can serve as a lifeline for community clinics, reliance on specific individuals can leave providers and staff vulnerable to staffing changes. One staff member (03) reported having a particular VA staff member “on speed dial” because “she takes it on and things get done.”

## DISCUSSION

In this qualitative study, we identified four themes that characterize the experiences non-VA primary care clinicians and staff have caring for rural veterans, including confusion, variability, and delays for VA administrative processes, clarifying responsibility for dual-user veteran care, accessing and sharing medical records outside the VA, and negotiating communication pathways between systems and providers. Bureaucratic pressures on community care clinics can create unnecessary care fragmentation and exacerbate inequities for rural veterans. Challenges navigating the VA system underpinned almost all participant experiences providing community care to rural veterans; workarounds were frequently described and helped to circumvent barriers.

The findings presented in this manuscript provide essential data to advance and expand conversations around how to improve access and quality of care for rural veterans. In a prior study, our research team utilized participatory systems mapping to visually describe complex dynamics underlying rural veteran access to care.^5^ The themes identified in this research drew from a broad participant base and align with themes identified in the current study. Multiple prior studies compare cost, quality, and veteran satisfaction with community care versus VA-provided care;^14,31–34^ however, the experiences of non-VA staff and clinicians who care for rural veterans are underrepresented in the research.^19,35–37^ Community care is known to increase access to primary care services, but community care and dual use by veterans introduces complexity for individual patients, and for VA and non-VA clinicians and staff providing care. ^21,38–42^

The challenge of care coordination for veterans, especially dual-user veterans, is well documented.^20,42–44^ While we set out to explore rural perspectives in order to identify potential unique experiences, our data depict challenges similar to those that urban veterans and urban community care providers have been found to face in prior studies.^45^ Our findings support prior research that rural veterans may carry an extra care coordination burden when using providers that sit outside of the VA.^43,46^ VA-supported care coordination efforts aim to mitigate this burden which can also reduce the burdens on clinicians and improve staff satisfaction.^47–51^ While there exists a body of literature exploring VA clinician experiences and challenges with care coordination across organizational boundaries,^15,45,46,52–55^ fewer studies explore the experiences of rural non-VA clinician and staff members.^18,19,56^ Our findings expand this conversation by identifying specific challenges that non-VA clinicians and staff have interfacing with and navigating VA systems to provide care to rural veterans. In particular, our findings illustrate that community care clinicians and staff identify systemic challenges with organizational processes and policy specific to the VA, rather than population and health challenges specific to veterans. With expanded community care network access under the MISSION Act, it is particularly important to design and implement solutions with non-VA stakeholder input.

Researchers emphasize the need to measure network adequacy by exploring differences by provider type, wait time, distance standards, provider ratios, or through qualitative assessments of experiences.^57^ While definitions of network adequacy may vary within and across insurance providers,^58,59^ evaluations of community care network adequacy appear limited.^57^ Our study provides important insights into community care network provider experiences which may impact the willingness of community care clinicians to see veteran patients. While many of these challenges are not limited to caring for veterans, for example information exchange across EHRs ^60–62^ or navigating administrative procedures,^63–65^ our informants noted amplified difficulties in navigating VA systems and structures and raised concern about the resulting quality of care they were able to provide. The workarounds identified, and additional solutions, suggest potential interventions at the clinic, community, and policy levels which could help ensure a sustainable community care network for rural veterans. Thoughtful approaches for rolling out policies, such as the VA’s current EHR transition to CERNER, are intended to provide opportunities to help address and reduce the bureaucratic burden of interacting with the VA noted by our participants and to help maintain the community care networks. Moreover, efforts to help inform community care clinicians about policy changes and to proactively facilitate access to information may enhance patient experience and quality of community care.

Provision of care in rural settings requires team-based care both within an individual clinic and across organizational boundaries. Processes that measure and improve interpersonal and interorganizational communication and shared understanding of goals, such as relational coordination, have been shown to improve team performance in various health care arenas including within the VA.^66–69^ However, these strategies have yet to be implemented across the VA/non-VA clinic boundary. Pilot activities to test these strategies in rural areas with participants inside and outside of the VA could be a valuable first step in reducing care fragmentation across VA and non-VA providers.

## Limitations

Our study has three main limitations. First, participants’ geographic distribution was limited to Oregon, Washington, and Idaho. Further inquiry into challenges experienced in other regions of the USA as well as to compare and contrast differences in rural versus urban settings could deepen our understanding of applicability and scalability of findings and inform future interventions. Second, interviews occurred concurrent with the COVID-19 pandemic, which may have impacted sampling and participant perceptions. Finally, because recruitment materials emphasized exploring participant experiences providing care for rural veterans, participants who have experienced more challenges providing this type of care may have self-selected. Despite these limitations, participants represented a diverse population of clinic roles, viewpoints, and experiences caring for rural veterans and study findings offer new insight into the experiences of clinicians and staff members providing community care to rural veterans.

## CONCLUSIONS

Our research indicates that clearer pathways of communication, streamlined or demystified administrative processes, access to records, and clarification of roles may help community care clinicians and staff care for rural veterans. Reducing these barriers for rural non-VA clinicians and clinic staff may help with retention of the providers in the community care network, potentially maintaining or expanding current access to care as intended by policies such as the 2019 MISSION Act. While clinic-level interventions (e.g., policy outreach, direct point of contact) may be important, changes through policy that support structural modifications that improve the ability of community care providers to interact with VA structures may have impacts that benefit veterans and all who provide their care. By illustrating the bureaucratic burden of interacting with the VA and highlighting workarounds that rely on trial and error or individual efforts, this research may inform program development for VA clinician and clinic staff training and resources, VA policy design, and cross entity relationship facilitation.

## Data Availability

The data that support the findings of this study are available from the corresponding author, MP, upon reasonable request.
